# Health Care Costs attributable to Hospital-diagnosed Back Pain: A Longitudinal Register-based Study of the Danish Population

**Published:** 2014-02-26

**Authors:** Rikke Søgaard, Jan Sørensen

**Affiliations:** 1 CFK - Centre for Health Services Research (CAST), University of Southern Denmark, JB, Odense, Denmark

**Keywords:** back pain, population norms, reference values, attributable costs, cost analysis

## Abstract

**Background:** Back pain is one of most frequent musculoskeletal conditions with enormous impact to health care systems and society. Analytical studies that guide the management of this disease are strongly needed, but there is a lack of cost estimates for the attributable cost of severe or chronic back pain in particular.

**Objective:** The objective of this study was to estimate the health care costs attributable to hospitaldiagnosed back pain across strata of age-, gender- and diagnostic entity.

**Methods:** All adult Danes (N=4.3 million) were included in this longitudinal, controlled register-based study. One-year prevalence was defined according to a previously published and validated algorithm, which was applied to the Danish national patient registry. Data from other relevant health service use registries was appended along with data from the national cause of death registry in order to calculate cost rates per life year (2011 price year). The attributable health care cost was defined as the absolute difference in cost rates between individuals with versus individuals without hospital-diagnosed back pain, whereas the ratio between the two groups was used for the reporting of reference values.

**Results:** The health care costs attributable to hospital-diagnosed back pain were estimated at Danish Crowns (DKK) 22,700 per year for the youngest age strata (16-24 years) and increased up to DKK 72,700 per year for the oldest age strata of males (>85 years). Hospital admissions and outpatient visits accounted for the majority of these costs. The ratio of health care costs for individuals with versus individuals without the condition ranged from less than 1 to almost 6, depending on the type of service use, age and gender.

**Conclusion:** At the disease stage where back pain leads to contact with specialised health care, diseased individuals appear to use on average three times more health care than non-diseased individuals. This study provides detailed reference values, which can be used to inform health economic models.

## 1. Background

Back pain is the most frequent of the musculoskeletal diseases and has enormous consequences to the individual and to society.[1] Not only does it suppress the health-related quality of life of the individual; often, it also prevents the individual from leading a productive work and leisure life. For the subgroup of individuals where the condition becomes long lasting, it has been shown that the rate of return to work quickly decreases and approaches zero after 2 years of sick leave.[2] This is obviously a prospect that the health care system should seek to prevent and thus it can be expected that health care use increase with the duration of symptoms.

The literature on the effect of various strategies for treating severe back pain is vast and recently a number of health economic studies have emerged to assess the cost effectiveness of treatment strategies.[3] These economic evaluations have primarily been conducted alongside clinical trials although it has been suggested that the follow-up periods of clinical trials are too short to observe the manifestation of all effects and that study populations are highly selected subgroups of target populations.[4] The role of health economic modelling thus seems to be underplayed in this particular area. This could be due to a lack of cost estimates that are based on comparative cost analysis.

The cost of illness literature contributes with general findings stating that a small proportion of back pain sufferers accounts for the largest share of the economic consequences due to the condition becoming chronic.[5] It also shows that about a quarter of total societal costs are due to health care use, depending on the survey context and population characteristics, but often it is not possible to discern the costs related to the subgroup of individuals with chronic pain. Both in a modelling context, and for the discussion of external validity of the existing and new trial-based economic evaluations, it is highly relevant to obtain reference values on the resource use of individuals with versus individuals without chronic back pain.

Besides being associated with severity or duration of the disease, the health care costs of individuals with back pain have been found to be associated with age and gender.[6,7] It is also well known that different diagnostic groups (often contrasted by specific and non-specific back pain) have different prognoses for symptom relief and therefore can be expected to demonstrate different cost rates.[8] Another caveat is that it is extremely complex to disentangle back pain costs from comorbidity costs that seem to constitute a major part of the total costs.[9] Altogether, these facts suggest that reference values for health care use across individuals with back pain should distinguish between age- and gender strata as well as diagnostic subgroups, and that the analytical approach should be designed to include comorbidity, if it can be attributed to back pain.

A further challenge that has great influence on the usefulness of cost reference values is the choice of strategy by which the study population is identified. If cost estimates are intended to inform economic models assessing the effect of hospital-based health care, study subjects obviously have to be sampled from a hospital setting. Moreover, given the severity of the condition and the typically right-skewed cost distribution, studies on cost rates have to pay particular attention to recall and non-response bias that may invalidate findings. In the present study, we circumvent these challenges by basing the identification of the study population and the measurement of resource use on registers that include the entire population, and in which there is generally no problem with completeness and recall.[10]

The objective of this study was to estimate the health care costs that are attributable to back pain in individuals who received hospital-based health care due to that condition, within the last year. We refer to this population as 'hospital-diagnosed' to emphasize that we deal with a specific subgroup of the broader entity of individuals with back pain, who may not seek specialised health care.

## 2. Methods

The study was designed as a longitudinal study of the entire Danish population above the age of 15 years (N=4.3 millions). The following section details the identification of the population, the measurement of individual cost categories, the analytical strategy and ethics.

### *Population Identification*

The national patient registry was used to identify individuals who have had contact to specialized health service during 2005 due to non-malignant, back pain. The identification strategy was based on a previously published algorithm that has been developed to identify pain patients from administrative data that include diagnostic codes of the International Disease Classification version 10.[8,11] The algorithm provides a classification into three diagnostic subgroups: pain in spinal disc disorders (M50 cervical disc disorders and M51 other intervertebral disc disorders), (other) specific back pain (M43 other deforming dorsopathies; M45 ankylosing spondylitis; M46 other inflammatory spondylopathies; M48 other spondylopathies; M49 spondylopathies in diseases classified elsewhere; M81 osteoporosis without pathological fracture and M82 osteoporosis in diseases classified elsewhere) and non-specific back pain (M47 spondylosis; M53 other dorsopathies, not elsewhere classified and M54 dorsalgia). Individuals who were not listed with any of these diagnoses were classified as not having hospital-diagnosed back pain.

### *Cost Parameters*

For the enitre population, records of health care service use from January 1, 2005 through December 31, 2005 were obtained from different registries: the national patient registry including all hospital use,[12] the national health insurance registry including all primary care service use[13] and the registry for prescription medication.[14] National average Diagnosis Related Group (DRG) tariffs were used as the costs of hospital service, fee-for-service tariffs were used to value primary care services and pharmaceutical sales prices were used for prescription medications. All costs are in Danish crowns (DKK) and refer to the price year of 2006. Cost rates were defined as costs per person year. For that purpose, we extracted person time from the cause of death registry,[15] which includes date of death.

### *Analytical Strategy*

The ratio of cost rates between individuals with and without back pain was used for the analysis of reference values. The ratio measure, which in epidemiological terms refers to the incidence rate ratio, was chosen in order to avoid the interpretation issues related to currency and price year.

In addition to the reference values that are reported in a table format for individual strata of age and gender, we assessed the absolute magnitude of costs attributable to hospital-diagnosed back pain, as well as the role of diagnostic subgroups and the distribution of costs across health care sectors, in graphical illustrations. The cost attributable to hospital-diagnosed back pain was defined as the cost difference between individuals with versus individuals without back pain. For ease of interpretation, the observed 1-year age strata were collapsed into 5-year age strata in the figures.

As the study is based on the entire population, there is no sampling uncertainty, and confidence intervals and statistical tests are therefore irrelevant.

*Ethics*

All data extraction and merging was conducted at the protected servers of Statistics Denmark, and the identity of individuals has at no point been revealed to the authors.

### 3. Results

The study population included all adult Danes above age 15 years (N=4.3 million). Of these, an overall proportion of 1.10% was observed to be in contact with the hospital-based health care system due to a back pain-related problem, during a 1-year observation period. This proportion varied from 0.31% in the youngest males to 2.02% in the female stratum between age 75 and 84 years. Table 1 further details the study population in terms of age, gender, prevalence and observed person time.

**Table 1. Population Characteristics: Number of Individuals and Person Years and 1-year Prevalence**

| | General Population | | Hospital-diagnosed Back Pain | | |
|---|---|---|---|---|---|
| | N | Person years | n | % of N | Person years |
| **Male age groups** | | | | | |
| 16-24 | 254730 | 254647 | 796 | 0.31 | 795 |
| 25-34 | 364270 | 364132 | 2569 | 0.71 | 2568 |
| 35-44 | 414061 | 413743 | 4564 | 1.1 | 4560 |
| 45-54 | 367947 | 367120 | 4464 | 1.21 | 4447 |
| 55-64 | 357354 | 355465 | 4003 | 1.12 | 3949 |
| 65-74 | 207630 | 204773 | 2405 | 1.16 | 2319 |
| 75-84 | 113381 | 109298 | 1448 | 1.28 | 1335 |
| >85 | 29920 | 27239 | 279 | 0.93 | 231 |
| All males | 2109293 | 2096418 | 20528 | 0.98 | 20205 |
| | | | | | |
| **Female age groups** | | | | | |
| 16-24 | 244971 | 244948 | 861 | 0.35 | 861 |
| 25-34 | 359881 | 359827 | 2556 | 0.71 | 2555 |
| 35-44 | 400613 | 400418 | 4355 | 1.09 | 4347 |
| 45-54 | 361476 | 360935 | 5072 | 1.4 | 5057 |
| 55-64 | 358037 | 356830 | 5465 | 1.53 | 5424 |
| 65-74 | 230420 | 228334 | 4290 | 1.86 | 4211 |
| 75-84 | 164552 | 160373 | 3322 | 2.02 | 3189 |
| >85 | 72178 | 66896 | 900 | 1.25 | 812 |
| All females | 2192128 | 2178561 | 26821 | 1.28 | 26457 |
| | | | | | |
| N | 4301421 | 4274979 | 47349 | 1.1 | 46662 |

In Figure 1, the average annual cost rates for individuals with and without back pain are illustrated. Cost rates include all health care costs, that is, primary health care, hospital-based health care and prescription medication provided by the primary care sector. As expected, costs increased steadily over lifetime, except for a minor peak for females during their fertile years. For males, there appeared to be a peak around age 75 years, but this could be due to low numbers of person years observed for individuals with hospital-diagnosed back pain after age 85 years (231 person years; Table 1). The scale of health care costs attributable to hospital-diagnosed back pain started at about DKK 22,700 per year for the youngest age strata (16-24 years) of both genders and increased up to DKK 72,700 per year for the oldest age strata of males (>85 years).

**Figure 1. Health Care Costs of Individuals with and without Hospital-diagnosed Back Pain: Annual Cost Rates**

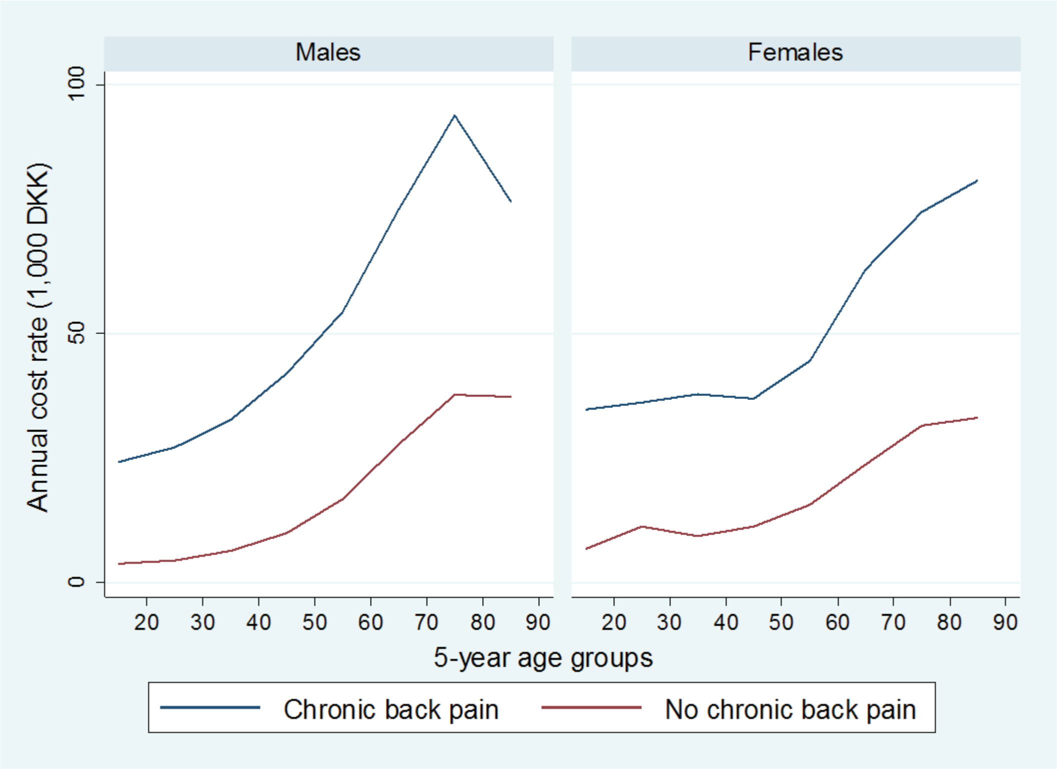

DKK=Danish crowns

*Health Care Costs Attributable to Hospital-diagnosed Back Pain*

The ratio of health care costs between individuals with versus individuals without hospital-diagnosed back pain was overall found to be 2.82 for males and 2.68 for females. These averages masked relatively wide variation across age strata but also across health care sectors, as illustrated in Table 2.

The ratio of health care costs between individuals with versus individuals without hospital-diagnosed back pain was almost 4 for young males, whereas it decreased throughout life end ended at about 2 for males aged >85 years, obviously due to the controls increasingly suffering from diseases as well. It should be noted that while the ratio was twice as high for the younger age strata, the absolute cost was significantly lower than that of the older age strata, because the baseline cost was relatively low for young people. A similar finding was observed with respect to the distribution across sectors. While the cost ratio for prescription medication was almost 6, this category accounted for only a minor proportion of the absolute costs of health care.

**Table 2. Reference Values for Health Care Costs attributable to Chronic Back Pain. Ratio of annual Cost Rates between Individuals with and without Hospital-diagnosed Back Pain**

| | General Practice | Private Practice Specialists | Private Practice Therapists | Other Primary Care | Prescription Medication | Inpatient | Outpatient | Total |
|---|---|---|---|---|---|---|---|---|
| **Male age groups** | | | | | | | | |
| 16-24 | 1.58 | 1.31 | 2.84 | 1.02 | 5.88 | 3.69 | 1.68 | 3.88 |
| 25-34 | 1.57 | 1.32 | 2.39 | 1.05 | 5.31 | 3.47 | 1.68 | 3.59 |
| 35-44 | 1.50 | 1.31 | 2.12 | 1.05 | 4.42 | 3.12 | 1.65 | 3.21 |
| 45-54 | 1.42 | 1.29 | 1.87 | 1.08 | 3.65 | 2.81 | 1.59 | 2.85 |
| 55-64 | 1.32 | 1.22 | 1.68 | 1.11 | 3.07 | 2.48 | 1.49 | 2.53 |
| 65-74 | 1.22 | 1.14 | 1.44 | 1.15 | 2.78 | 2.27 | 1.32 | 2.33 |
| 75-84 | 1.13 | 1.07 | 1.13 | 1.22 | 2.59 | 2.13 | 1.14 | 2.17 |
| >85 | 1.00 | 0.91 | 0.94 | 1.18 | 2.38 | 1.83 | 0.94 | 1.98 |
| All males | 1.34 | 1.20 | 1.80 | 1.11 | 3.76 | 2.73 | 1.44 | 2.82 |
| **Female age groups** | | | | | | | | |
| 16-24 | 1.49 | 1.30 | 2.55 | 1.13 | 4.71 | 2.95 | 1.92 | 3.20 |
| 25-34 | 1.47 | 1.25 | 2.35 | 1.13 | 4.11 | 2.82 | 1.79 | 2.93 |
| 35-44 | 1.45 | 1.25 | 2.06 | 1.16 | 4.04 | 2.84 | 1.76 | 2.89 |
| 45-54 | 1.42 | 1.23 | 1.86 | 1.19 | 3.52 | 2.70 | 1.70 | 2.67 |
| 55-64 | 1.39 | 1.21 | 1.72 | 1.24 | 3.20 | 2.54 | 1.62 | 2.54 |
| 65-74 | 1.35 | 1.13 | 1.56 | 1.31 | 3.02 | 2.42 | 1.53 | 2.45 |
| 75-84 | 1.31 | 1.07 | 1.43 | 1.41 | 2.89 | 2.37 | 1.41 | 2.38 |
| >85 | 1.25 | 0.91 | 1.45 | 1.59 | 2.92 | 2.30 | 1.28 | 2.39 |
| All females | 1.39 | 1.17 | 1.87 | 1.27 | 3.55 | 2.62 | 1.63 | 2.68 |

### *The Role of Diagnostic Subgroups and Distribution across Sectors*

Figure 2 illustrates the distribution of the cost burden on different health care sectors. As expected from the population identification, the hospital sector faced the greatest share of the excess demand of individuals suffering from hospital-diagnosed back pain. This was due to higher costs per service at the hospitals, as compared with e.g. pharmaceuticals, and to the structural fact that long lasting or more severe diseases were typically handled in the secondary health care sector.

**Figure 2. Sectoral Distribution of the Health Care Costs attributable to Hospital-diagnosed Back Pain**

DKK: Danish crowns

Figure 3 illustrates the role of diagnostic grouping on attributable costs, which was characterized by large variation both over age strata and between genders. It seemed that the two specific pain subgroups demonstrated the highest attributable costs until midlife, and that the non-specific pain subgroup showed increasing cost rates. Although this was not entirely clear, it suggests that different reference values may not necessarily be needed for the different diagnostic groups.

**Figure 3. Diagnostic Distribution of the Health Care Costs attributable to Hospital-diagnosed Back Pain**

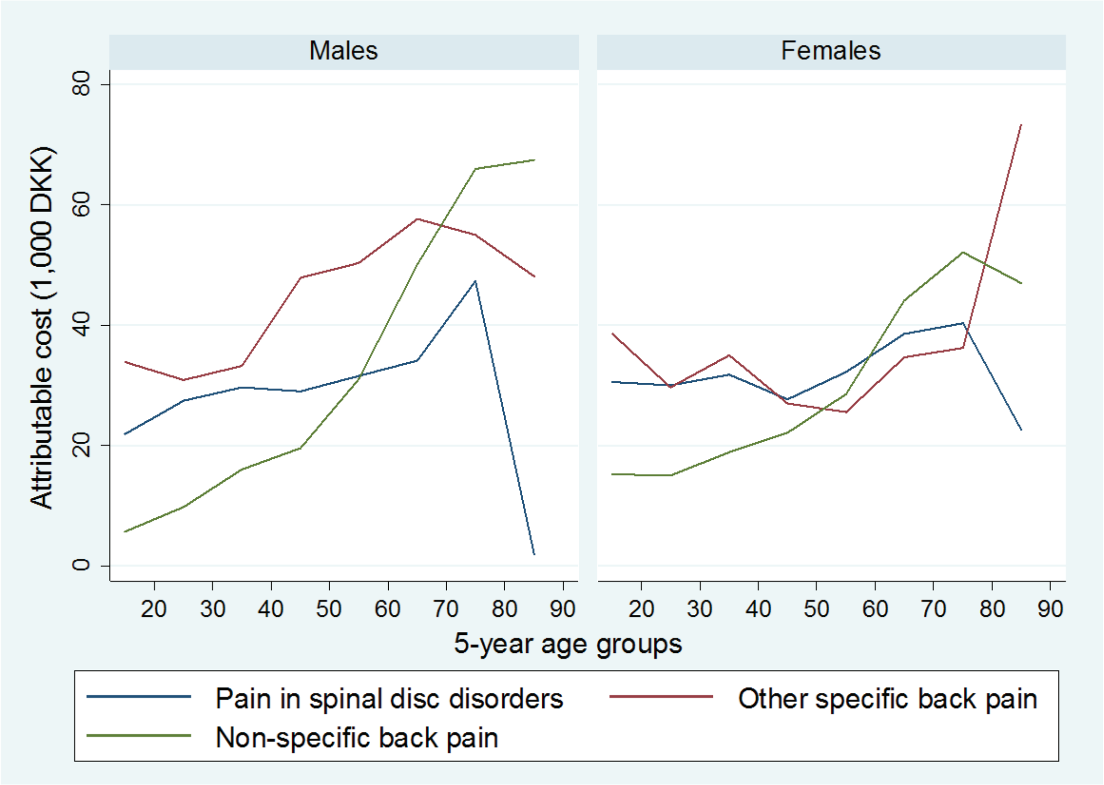

DKK: Danish crowns

## 4. Discussion

This study demonstrated that, at the disease stage where back pain leads to contact with specialised health care, diseased individuals use about three times more health care services than non-diseased individuals. These results can be used to inform health economic models assessing the cost effectiveness of existing or new technologies.

*Comparison with Existing Studies*

Few previous studies have been conducted within the subgroup of patients suffering severe back pain and there appears to be none within the specific target population of the present study.

In a recent study from the general practice setting in the United Kingdom, it was found that patients with chronic back pain demonstrate a factor 2 cost of matched controls without the condition.[16] This factor is marginally lower than that of the current study, but since the study did not the include inpatient service cost category, the results cannot be directly compared. Also, the matching procedure (based on age, gender and geography in terms of general practice affiliation) means that this study essentially analysed costs attributable to back pain when first adjusted for the other covariates, whereas the present study reports costs attributable to back pain (including any covariates of particular age- and gender profiles or geography).

Another study from the primary care setting origins from Sweden and includes only 302 patients.[17] The authors report that the composition of costs was distributed with about half of the health care costs related to inpatient and outpatient hospital service, which was then followed by use of physical and chiropractic therapy and pharmaceuticals, and a few inferior cost categories of diagnostic tests, paid home aid and orthopaedic aids. The fact that we observed a larger share of costs within hospital-based services thus suggest that the current population is in a more progressed disease state than a population identified in a primary care setting. This is supported by another recent study where it was concluded that there is a factor 2 difference in costs between patients sampled in primary and secondary care.[7]

There is one example of a study where patients were sampled from the waiting list for a hospital-based pain clinic. Kronborg *et al.* described the costs of chronic back pain patients in a non-controlled cohort of 204 patients observed for up to 9 years.[6] This study confirms the role of age and severity of symptoms in relation to total health care costs, whereas the authors did not find gender to be associated with cost. Finally, there is a large North American study of chronic pain sufferers enrolled in a particular health plan that estimates chronic back pain to account for a factor 2.3 increased health care.[5] This confirms the magnitude found in the current study despite the different structural characteristics of health care systems in North America and Denmark.

### *Study Strengths and Weaknesses*

The main strength of the study relates to the controlled design, including the whole population, and to the use of registries that circumvent issues related to non-response and to recall problems. A further strength is the identification of the population, which is based on a previously validated algorithm[8,12] with a clear-cut definition in terms of the International Disease Classification. This is an important strength for the usability of the provided reference values in health economic models.

The main weakness of the study relates to the quantification of the cost parameter and whether we have actually been able to capture all relevant resource use. The current study included only cost categories that were available in national registries and that means that we may have omitted relevant costs. In a British clinical trial participants were asked about their use of over-the-counter medication as well as services from complementary practitioners.[19] It can be roughly calculated from their report that the magnitude of visits to complementary practitioners amount to 2% to 4%, whereas over-the-counter costs amount to less than 1% of total health care costs. This suggests that we have underestimated the total cost due to having omitted these cost categories. On the other hand, it is not our impression that their magnitude will fully carry over to the difference between individuals with versus without chronic back pain. A related issue is the valuation of services provided in the primary care sector, which was conducted using the fee-for-service tariffs used for remuneration. However, for general practitioners we have underestimated the average cost, as they receive capitation payment corresponding to about one-third of their total remuneration. Finally, it may have been relevant to also include social services such as home care and nursing home placement.

### *Limitations and Perspectives*

It is important to emphasize that the current results have external validity in target populations that match the study population only. This means that the results can be used for a population that is referred for hospital-based care as opposed to care managed by general practice.

In terms of future perspectives, it would be interesting to estimate the lifetime costs per individual of a certain age and gender strata using the life table method, as was recently demonstrated in a previous article in this journal for the general population.[20] Given that the current data are based on 1-year prevalence, this would imply an assumption about constant resource use over the course of the natural disease history, which seems to be inappropriate. It would therefore be interesting for future studies to base the identification of the study population not just on period prevalence but on real incidence data, in order to generate lifetime costs.

The present reference values for the cost load attributable to back pain have been developed to provide researchers who want to model the costs of back pain in, e.g. a cost of illness or a cost-effectiveness study with load factors that are not directly dependent on price levels, currency or price year. In many settings, the average use of health care is available for the general population but not for disease-specific subgroups. Our estimates can be used as multipliers for reference values of the general population to provide a cost estimate for individuals with back pain. As a final remark, it should be noted that back pain costs reach far broader than just health care costs, and, in particular, costs due to productivity losses could be equally relevant in model studies.

## 5. Conclusion

The health care costs of individuals with hospital-diagnosed back pain are substantially higher than those of individuals without such a condition. There appears to be only moderate variation between main diagnostic subgroups, whereas there is substantial variation across age strata. The impact of back pain on the cost ratio of health care costs between individuals with and without diagnosis is highest for the younger as opposed to the older age strata, particularly for men. Detailed reference values for the cost ratios between individuals with and without hospital-diagnosed back pain are provided and may inform future health economic models.

### Declaration of Competing Interests

The authors declare that they have no competing interests.

### Acknowledgements

This work was part of the CESpine project (www.cespine.org), which is financed by the Danish Strategic Research Council (grant 2142-08-0017).
